# Spatial association network of economic resilience and its influencing factors: evidence from 31 Chinese provinces

**DOI:** 10.1057/s41599-023-01783-y

**Published:** 2023-06-05

**Authors:** Huiping Wang, Qi Ge

**Affiliations:** Western Collaborative Innovation Research Center for Energy Economy and Regional Development, Xi’an University of Finance and Economics, 710100 Xi’an, China

**Keywords:** Economics, Development studies

## Abstract

The spatial correlation pattern of economic resilience is an important proposition for China’s sustainable economic development. This paper measures the economic resilience of 31 provinces in China from 2012 to 2020, and explores the spatial correlation of economic resilience from the overall, group and individual perspectives and its influencing factors. The results show that first, a tightly ordered hierarchy of economic resilience formed in each province of China after 2016. Among them, Jiangsu, Shandong, Guangdong, Hubei, and Shaanxi are the most important clustering points and radiation centers in the spatial correlation framework of economic resilience. Second, being adjacent to marginal and core provinces will maintain the province’s centrality index category to the greatest extent, while being adjacent to sub-core and general provinces leads the province to gain more opportunities for upward transfer. Third, the essence of the interprovincial economic resilience subordination linkage in China is manifested in the aggregation of city clusters or economic circles. The northern economic resilience linkage system with the Bohai Rim as the core contains more provinces but is less stable. Provinces located in the Yangtze River Delta region are the opposite. Fourth, the proximity of geographical location and the difference in human capital level drive the formation of spatial association networks, while the difference in external openness and the difference in physical capital inhibit the formation of networks.

## Introduction

Recently, with the new coronavirus epidemic still ravaging the world, high inflation levels in major economies, and the Russian-Ukrainian conflict further exacerbating the energy and food crises, the world has been facing the most serious systemic crises since the end of the Second World War. Against the backdrop of the impact of these unexpected factors, this coincides with the fact that China’s economy is in a critical period of transforming its development mode, optimizing its industrial structure and shifting its growth momentum. Enhancing economic resilience has become the key for China to resist external risks and promote high-quality economic development (Hu et al., 2022). However, due to the vast size of China, there are significant differences among regions in terms of geographic location, resource endowment, and economic development, which leads the economic resilience of each region to vary greatly in the face of external shocks: some regions have a less pronounced recession after a shock and can recover quickly, reflecting better economic resilience, while other regions have a significant economic decline or persistent recession after a shock reflecting weaker economic resilience. Moreover, interprovincial economic activities are inextricably linked, and the connection between economic resilience is no longer limited to geographical proximity but gradually takes on a network form. Against this background, it is of great significance to precisely characterize the network structure of economic resilience and its evolution, to clarify the role of each province in the spatial association network and to reveal the influencing factors of the network such that the ability of each region to cope with external shocks can be enhanced and the sustainable development of China’s economy can be promoted.

In recent years, economic resilience has gradually become a hot topic of research globally (Hu and Yang, 2019; Du et al., 2020; Cheng et al., 2022). The concept of “resilience” was first applied by Holling (1973) in the field of systems ecology and later introduced to the economic field by Reggiani et al. (2002). Martin (2012) proposed four research dimensions of regional economic resilience, namely, the ability to resist and absorb shocks, the speed and extent of recovery aftershocks, the ability to reintegrate internal resources and adjust the economy structure to the new external environment aftershocks, and the ability to create new paths for the regional economy. In addition, many other scholars have provided definitions of economic resilience from different perspectives, but in general, its connotation is basically the same; this connotation includes two aspects: on the one hand, emphasizing the ability of a region to resist and adapt after a shock, and on the other hand, emphasizing the ability of the entire regional economy to adjust and transform itself and achieve a “path breakthrough” (Boschma, 2014; Tan et al., 2020; Hu et al., 2022; Xie et al., 2022). In the pre-shock period, the regional economic system generates the most direct and mechanical response to the shock to keep the economic function and structure unchanged, concentrating on the resistance of the regional economic system. In the middle of the shock, the speed and extent of economic recovery is accelerated by means of rapid and diversified resource factor mobilization, which may eventually lead to a leap to a new equilibrium state of the economy (Martin and Sunley, 2014). Scholars have conducted a series of studies on ways of measuring economic resilience and its influencing factors. The current methods for measuring economic resilience are mainly the sensitive index method and the composite index method. According to the sensitive index method, a variable directly reflects economic resilience. Examples include unemployment and GDP (Davies, 2011; Brakman et al., 2015; Tan et al., 2020), employment growth rate (Giannakis and Bruggeman, 2017), employed population (Chen, 2022), and economic value-added ratio (Sun et al., 2022). However, the sensitive index method has some drawbacks. For example, the method is only applicable to larger economic shocks and disturbances, the contemporaneous measurement is not sufficiently accurate, and the selection of sensitive variables is narrow. Therefore, the comprehensive index method, which has more information describes economic resilience based on different dimensions, such as resistance, renewal, repositioning and resilience, impact response, organizational adjustment, independent innovation, and reconstruction. Most of the methods include the following indicators: GDP growth rate, GDP per capita, industrial structure diversification, foreign investment dependence, unemployment rate, investment in fixed assets, ratio of deposit and loan balances, advanced level of industrial structure, and education expenditure (Cowell, 2013; Bristow and Healy, 2014; Heeks and Ospina, 2019; Alessi et al., 2020; Hu et al., 2022; Wang et al., 2022). The resilience of regions in the face of economic shocks is determined by the influence of a complex set of factors that together determine the vulnerability of regions to economic crises and the ability of the system to sustain, adapt and recover. Examples include industrial structure (Lagravinese, 2015; Martin and Sunley, 2014; Xiao et al., 2018; Martini, 2020; Mai et al., 2021), foreign investment (Tan et al., 2017), human capital (Crescenzi et al., 2016), technological innovation (Huggins and Thompson, 2015; Bristow and Healy, 2018; Xu and Deng, 2020), demographic structure (Dube and Polese, 2016; Xie et al., 2022), urbanization (Brakman et al., 2015), and COVID-19 (Wang et al., 2022).

A view originating from the “growth pole theory” suggests that the evolutionary space of an economic system starts with one or more growth cores that spread through different paths and eventually affect the entire regional economic development (Feng et al., 2023). In this evolutionary process, key elements of economic development, such as technological innovation, human capital, and financial resources, integrate and complement each other with agglomeration and spillover effects, and regions evolve toward a symbiotic economic system with high resilience as its essential characteristic, thus enhancing the ability to resist external shocks (Shi et al., 2021; Liu et al., 2022; Yi et al., 2023). Although there are certain differences in economic resilience in different regions, the first law of geography holds that any economic activity is spatially correlated. Some scholars have used exploratory spatial data analysis (ESDA) and spatial econometric models to study economic resilience, arguing that economic resilience has significant spatial dependence and agglomeration characteristics in geographic space, i.e., the economic resilience of a region is positively influenced by the economic resilience of neighboring regions (Chacon-Hurtadoa et al., (2020); Wang and Zhu, 2021; Cheng et al., 2022). However, there are generally still relatively few studies on the spatial correlations of economic resilience, and hence, there are various shortcomings. First, economic resilience is mainly explained or measured from a static perspective, and due to the interconnectedness of regions, the failure of one economic system will be transmitted to other systems when it is subjected to external shocks. Similarly, the readjustment of industrial structure after a shock does not involve only one region but has a certain spatial correlation. Second, the revealed spatial correlation and spillover effects of economic resilience only consider geographical proximity, while economic resilience also generates spillover effects in non-neighboring regions. Third, the structural patterns and spatial clustering of the spatial correlations of economic resilience are not further revealed, and in traditional models, spatial factors are only considered in terms of their quantitative effects, while spatial correlations cannot be revealed.

As mentioned above, this means that economic resilience as a system property is neither equivalent to an arithmetic average of the resilience of its individual members nor a mere combination of the resilience of its members. Therefore, to understand economic resilience more rationally, we need to model the interactions of individual members under the influence of ecological, educational, and technological factors (Hepfer and Lawrence, 2022). Taking the complex system perspective implies that systemic properties, such as resilience, need to be understood as emerging from the interaction of individuals. Hence, we must develop a bottom-up perspective for economic resilience, starting from the micro level rather than from the macro, or systemic level (Schweitzer et al., 2022). This is in line with the methodological principles of analytical sociology, aiming at explaining macro-social phenomena from the micro-processes that generate them (Flache et al., 2022). Individual members, as nodes in a network of economic relationships, have different characteristic attributes such as status, role, primary connection, and clustering, and such attributes depend on the attributes of other nodes or sets of nodes and they change over time. Therefore, our study explores the spatial association structure of economic resilience in China and its influencing factors using social network analysis (SNA). This paper contributes marginally to the literature in the following areas.

1. We construct a comprehensive index evaluation system of economic resilience and measure the economic resilience of 31 provinces in mainland China using the entropy-TOPSIS method to explore the more typical and exemplary economic resilience of China from an interprovincial perspective.
2. Based on the relational data and network perspective, we construct the spatial association network of the economic resilience of 31 provinces based on the modified gravity model, portray the structural form of the spatial relationship of economic resilience, and study the association structure and influencing factors of economic resilience within the nation from the interprovincial perspective.
3. The structural pattern of interprovincial economic resilience is identified and visualized by means of SNA at four levels: overall, individual, interindividual affiliation and clustering. A quadratic assignment procedure (QAP) is used to analyze the influence of different factors on the spatial association network of economic resilience, avoiding the problem of multicollinearity.
4. We use the entropy-TOPSIS method to obtain the centrality index by aggregating three centralities and, for the first time, explore the spatial transfer characteristics of node centrality by using spatial Markov chains. The concept of subordination is proposed to measure the characteristics of node linkage. The subordination association network is derived, and the structural characteristics of the spatial subordination association network are described in two dimensions: overall and local, which is an effective supplement to the existing methods.

## Methods

### Indicators of economic resilience

With reference to the measurement results of economic resilience by domestic and foreign scholars and the actual economic structure characteristics of China (Hu et al., 2022; Wang et al., 2022), we select 13 indicators from the three aspects of economic resistance, economic adaptation, and economic transformation to build a comprehensive evaluation system of economic resilience. Based on the existing research (Wang et al., 2022; Xie et al., 2022), the entropy-TOPSIS method (see Appendix A), which is objectively assigned and does not cause data information loss, is used to determine the weights of economic resilience indicators for each province. The specific evaluation indicators are presented in Table 1.

**Table 1:** Evaluation indicators of economic resilience.

Target layer	System layer	Index layer	Attributes
Economic Resilience	Resistance	GDP growth rate	+
		GDP per capita	+
		Industrial structure diversification level	+
		Foreign investment dependence	−
		Unemployment rate	−
	Adaptation	Total factor productivity	+
		Investment in fixed assets	+
		Ratio of deposit and loan balances of financial institutions	+
		Fiscal expenditure as a proportion of GDP	+
	Transformation	Advanced level of industrial structure	+
		Science and technology expenditure	+
		Number of patents granted	+
		Education expenditure	+

### Improved gravity model

The current methods for describing spatial correlations are mainly based on vector autoregressive models and gravity models. Since the vector autoregressive model cannot portray the evolution of the spatial association network and is too sensitive to the choice of lag order, this paper adopts the improved gravity model to construct the spatial association network of interprovincial economic resilience in China. The product of GDP and economic resilience is used to represent the quality of economic resilience of the province, and the modified gravity model is shown in Eq. (1).

1 $$Y_{ij} = k_{ij}\frac{{\sqrt {G_i \cdot {\mathrm{ER}}_i} \sqrt {G_j \cdot {\mathrm{ER}}_j} }}{{d_{ij}}},k_{ij} = \frac{{{\mathrm{ER}}_i}}{{{\mathrm{ER}}_i + {\mathrm{ER}}_j}}$$

where *Y*_*ij*_ denotes the gravitational force of province *i* on province *j*; ER_*i*_ and ER_*j*_ denote the economic resilience indexes of province *i* and province *j*, respectively; *G*_*i*_ and *G*_*j*_ denote the regional GDP of province *i* and province *j*, respectively; *d*_*ij*_ denotes the geographical distance between province *i* and province *j*; and *k*_*ij*_ characterizes the contribution of province *i* to the gravitational force of economic resilience between provinces *i* and *j*. Based on Eq. (1), we calculate the gravity matrix of economic resilience in China. In the matrix, each row’s mean value is set as the threshold value, gravitational forces above that value are recorded as 1, and gravitational forces below that value are recorded as 0. In turn, the binary matrix is obtained, as shown in Eq. (2).

2 $$Y\left( {i,j} \right) = \left\{ {\begin{array}{*{20}{l}} {0}&{y_{ij} \,<\, \overline y } \\ {1}&{y_{ij} \,\ge\, \overline y } \end{array}} \right.$$

### Characteristic indicators of the network

#### Overall characteristic indicators

The four indicators of network relevance, network density, network hierarchy, and network efficiency are used to describe the overall network characteristics of economic resilience. Network relevance refers to whether two nodes in a spatial association network are able to establish a connection with each other. The more unreachable provinces in the network are, the smaller the network relevance is. In this paper, we measure network relevance in terms of the total number of associated relationships in the network. Network density is defined as the ratio between the actual and maximum number of relationships and is a measure of how closely connected the nodes are. The greater the network density is, the stronger the ties between provinces are. Network hierarchy refers to the extent to which the nodes of a directed network are asymmetrically accessible to each other. The larger the degree of hierarchy is, the more unidirectional relationships there are in the network and the more distinct the hierarchical structure between individual provinces. Network efficiency reflects the efficiency of links between provinces. The lower the network efficiency is, the more stable the network is.

#### Individual characteristic indicators

In a network with interacting members, the status characteristics of nodes are the key to deeply explore the nature of the network and grasp the center of gravity of the network. Degree centrality, betweenness centrality and closeness centrality are used to characterize the importance of network nodes. Using the number of connections, degree centrality can be used to measure the position of each province in the network. If the degree centrality of a province is higher, it means that the province has more connections with other provinces, and the more likely the province is located at the center. Among them, degree centrality is also divided into point-out degree, which refers to the number of relationships that the province actively sends to other provinces, and point-in degree, which refers to the number of relationships that the province passively receives from other provinces. Betweenness centrality reflects the extent to which a province controls the relationships between other provinces, i.e., the extent to which it is in the “middle” of other provinces. The higher the betweenness centrality, the greater the province’s ability to control the flow of economic resources among other provinces, and the more likely it is to be located at the center. Closeness centrality describes the degree to which a province in the network is not controlled by other provinces in the process of economic resource connection. A province with a higher closeness centrality will have more links with other provinces and be the central actor.

#### Spatial subordination association structure

The number and complexity of the relationships in social networks are many, and many of them are not solid or in a secondary subordinate position. Therefore, simplifying the relationships in the network and extracting the main connection “skeleton” is an important problem to be solved. In this paper, we propose the concept of subordination to measure the main direction of interprovincial linkages to clearly and concisely determine interprovincial dependency and independence, and on this basis, we divide the network into chunks and subordinate groups.

The definition of dependency is shown in Eq. (3). The denominator represents the sum of the part of the mutual gravitational force generated by province $i$ and all other provinces, which is contributed to by other provinces. The numerator represents the part of the gravitational force generated by province $i$ and generated by province $j$ that is contributed by province $j$. The division of the two indicates the degree of attraction of province $i$ to province $j$. The larger the value is, the higher the subordination of province $i$ to province $j$. The affiliation matrix is constructed by taking the affiliation value of province $i$ to all other provinces as the horizontal row of the relationship matrix. The threshold value of each row is set to 0.2, and a subordination degree greater than 0.2 indicates the existence of a subordination relationship between provinces and is recorded as 1. Conversely, if the subordination degree is lower than 0.2, it is recorded as 0. Finally, the spatial subordination correlation network is established, with the arrow in the directed network indicating subordination and the node designated by the arrow indicating the province receiving the subordination relationship.

3 $$S_{ij} = \frac{{y_{ji}}}{{\mathop {\sum}\nolimits_{j = 1}^n {y_{ji}} }}$$

#### Block model

The block model analysis can examine the development of the spatially linked network of economic resilience from a new dimension, reveal the internal structural state, find the number of plates in the network and the provinces contained in each plate, and then analyze the relationships and connections between the plates. Therefore, we use the block model to classify the roles of blocks in spatially linked networks into four types: benefit plate, overflow plate, bilateral spillover plate and broker plate.

#### QAP analysis

Since all variables in the study of factors influencing the spatial association network of economic resilience are relational data and since these relational data may be highly correlated with each other, they cannot generally be tested using traditional econometric methods. QAP is a nonparametric method that requires no assumption of independence among independent variables and is more robust than parametric methods. Therefore, this paper uses the QAP procedure to discriminate and extract the influencing factors of the spatial association network and fit regressions on their degree of action. In this paper, five variables closely related to economic resilience are selected as influencing factors, and the model is set as in Eq. (4).

4 $${\mathrm{ER}} = f\left( {{\mathrm{GD}},{\mathrm{OPEN}},{\mathrm{DE}},{\mathrm{HC}},{\mathrm{PCS}},{\mathrm{TI}}} \right)$$

where GD is geographical proximity, OPEN is the level of openness, DE is the digital economy index, HC is human capital, PCS is the physical capital stock, and TI is technological innovation capacity.

### Spatial Markov chain

Markov chains can effectively portray the evolutionary process of things, discretize the research object into different state levels, and calculate the probability of its state shift. From the network perspective, the stability of the centrality of economic resilience of each province can be easily determined. The spatial Markov chain, on the other hand, determines the neighborhood states by the product of the spatial weight matrix and spatial lag operator and is used to explore the influence of different neighborhood economic resilience levels on the evolution of resilience levels in the region.

### Data source and processing

The sample period of this study is determined from 2012 to 2020, and 31 provinces of China are used as the research objects. The data are obtained from the China Statistical Yearbook, China Industrial Statistical Yearbook, China Science and Technology Statistical Yearbook, China High Technology Industry Statistical Yearbook, and EPS Database. Foreign investment dependence is expressed as the ratio of actual foreign investment utilization to GDP. Total factor productivity is calculated by the DEA-Malmquist method, with the real GDP of each province as the output variable and it is adjusted to constant prices in 2012 according to the GDP deflator. The input variables are labor input and capital stock, with the number of employed people as labor input and the amount of real social fixed asset investment as a proxy for capital input. Industrial structure diversification is measured by calculating the diversification index (DIV), which is based on the HHI index, using an entropy measure and is essentially the negative sum of the natural logarithm product of the employment component in each industry and these proportions, and it is calculated as shown in Eq. (5), where *e*_*is*_ is the number of people employed in industry *s* in province *i* and *e*_*i*_ is the sum of the total number of people employed in all industries in province *i*. A larger DIV indicates a higher degree of relative diversification. The high-grade industrial structure (HIS) is reflected by the difference in the weight assigned to primary, secondary and tertiary industries, and the calculation formula is shown in Eq. (6), where ISG_1*i*_, ISG_2*i*_, and ISG_3*i*_ denote the ratio of primary, secondary, and tertiary industries in province *i* to the GDP of the sample provinces, respectively. The closer HIS is to 0, the lower the degree of sophistication. The proportion of total imports and exports to GDP is used to measure the degree of openness. The digital economy index is obtained using the ratio of information technology employees, the ratio of internet access users and the financial inclusion index after standardization, and it is calculated by equal weighting. Human capital is measured using the average number of years of education of the population in each province. Physical capital stock is calculated by the fixed asset investment and perpetual inventory method. Technological innovation capacity is measured using the full-time equivalent of R&D personnel. The geographic adjacency matrix is denoted as 1 if two provinces are adjacent to each other; otherwise, it is denoted as 0.

5 $${\mathrm{DIV}}_i = - \mathop {\sum}\limits_{s = 1}^s {\left( {\frac{{e_{is}}}{{e_i}}} \right)} \ln \left( {\frac{{e_{is}}}{{e_i}}} \right)$$

6 $${\mathrm{HIS}}_i = \left( {{\mathrm{ISG}}_{1i} \times 1 + {\mathrm{ISG}}_{2i} \times 2 + {\mathrm{ISG}}_{3i} \times 3} \right)/3$$

## Results and discussion

### Provincial differences in China’s economic resilience

The above method was used to obtain the economic resilience of each province in China in 2020. The results are shown in Table 2. The economic resilience ranges from 0.217 to 0.647, with a mean value of 0.346 and a standard deviation of 0.283. Overall, the economic resilience level of each province in China varies greatly and has not yet achieved synergistic development. The economic resilience shows a step distribution feature from the east, central, northwest to northeast. At the subsystem level, it is worth noting that some underdeveloped provinces have higher-than-average resistance levels due to the low foreign capital dependence of these small economies, which have relatively outstanding advantages in terms of high mobility and structural flatness. The level of conversion power is higher than the average value of only 10 provinces, and all of them are coastal provinces and central developed provinces. This indicates that China’s innovation environment and science and technology investment vary greatly, and there is still a long way from synergistic development. Beijing, Shanghai, Jiangsu, Shandong, Guangdong and Sichuan have outstanding performance in three subsystems, and there is no pattern of one system being below the average.

**Table 2:** Economic resilience of China’s provinces in 2020.

City	Economic resilience	Resistance	Adaptation	Transformation
Beijing	0.647	0.268	0.195	0.184
Tianjin	0.217	0.132	0.041	0.043
Hebei	0.355	0.157	0.152	0.046
Shanxi	0.331	0.17	0.138	0.024
Inner Mongoria	0.314	0.153	0.14	0.021
Liaoning	0.264	0.153	0.079	0.032
Jilin	0.296	0.176	0.095	0.025
Heilongjiang	0.235	0.081	0.12	0.034
Shanghai	0.454	0.18	0.163	0.111
Jiangsu	0.555	0.267	0.185	0.102
Zhejiang	0.43	0.242	0.106	0.082
Anhui	0.343	0.144	0.139	0.06
Fujian	0.423	0.227	0.165	0.031
Jiangxi	0.22	0.083	0.098	0.04
Shandong	0.43	0.213	0.142	0.075
Henan	0.311	0.156	0.101	0.054
Hubei	0.366	0.209	0.103	0.053
Hunan	0.261	0.118	0.094	0.049
Guangdong	0.62	0.263	0.172	0.185
Guangxi	0.342	0.229	0.075	0.038
Hainan	0.296	0.197	0.049	0.05
Chongqing	0.227	0.129	0.065	0.033
Sichuan	0.421	0.179	0.188	0.054
Guizhou	0.234	0.161	0.038	0.035
Yunnan	0.304	0.188	0.083	0.033
Tibet	0.254	0.145	0.091	0.018
Shaanxi	0.354	0.17	0.158	0.027
Gansu	0.334	0.191	0.113	0.03
Qinghai	0.274	0.168	0.097	0.009
Ningxia	0.244	0.135	0.099	0.011
Xinjiang	0.369	0.22	0.134	0.015

### Analysis of the spatial association network

**Fig. 1:**
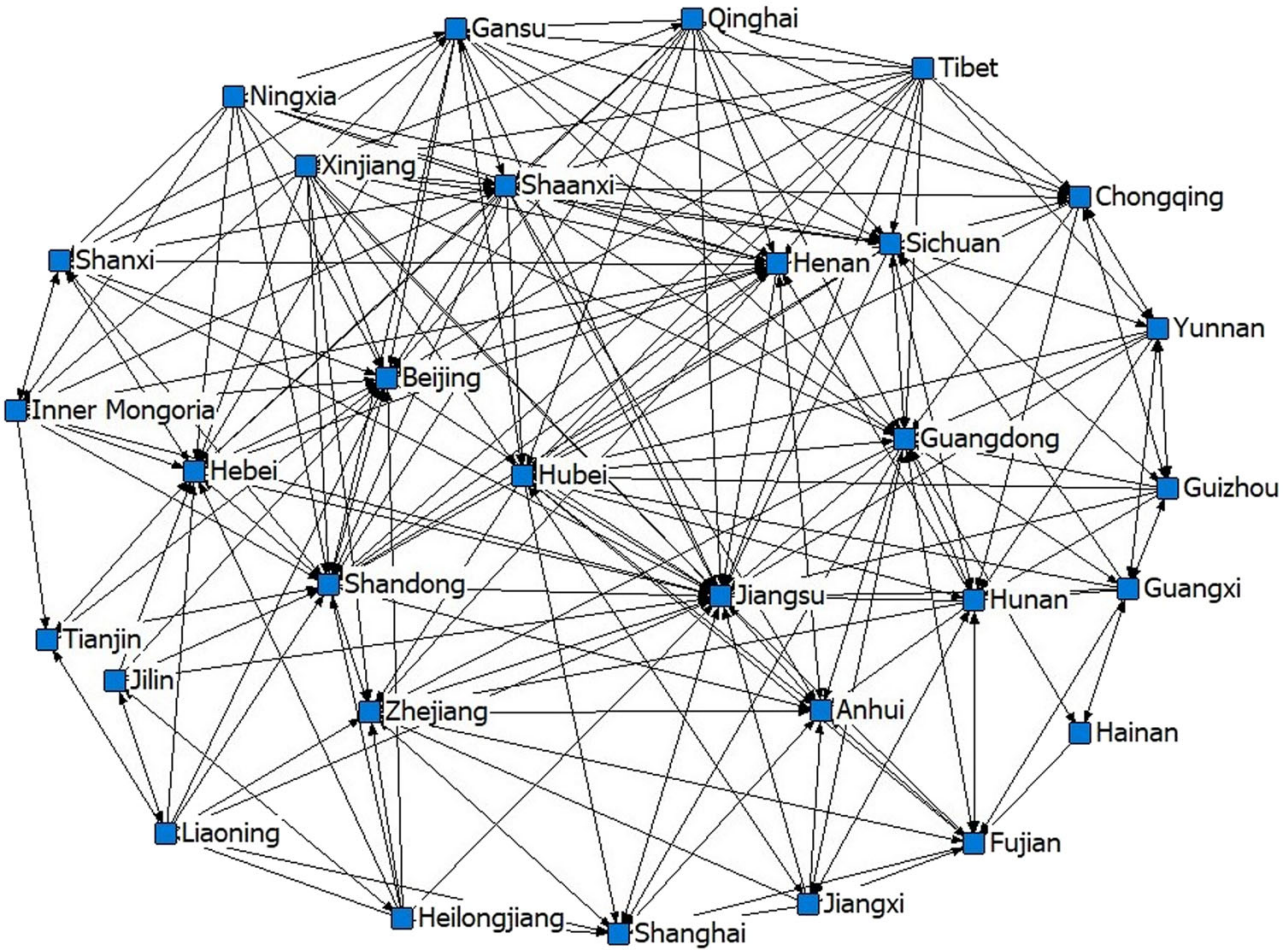
Spatial association network of economic resilience in China in 2020. The blue nodes represent provinces, and the directed line segments indicate outgoing or incoming association relations.

This paper determines the spatial correlations of the economic resilience of 31 Chinese provinces in 2012–2020. The network diagram drawn from this can examine and compare the degree of spatial correlation among provinces in terms of the overall, individual, subordination and subgroups. Due to space limitations, this paper only draws the network diagram for 2020 using the UCINET6 visualization tool Netdraw. As presented in Fig. 1, the spatial correlation of economic resilience among Chinese provinces shows a network structure with a multithreaded pattern.

#### Overall network characteristics

**Fig. 2:**
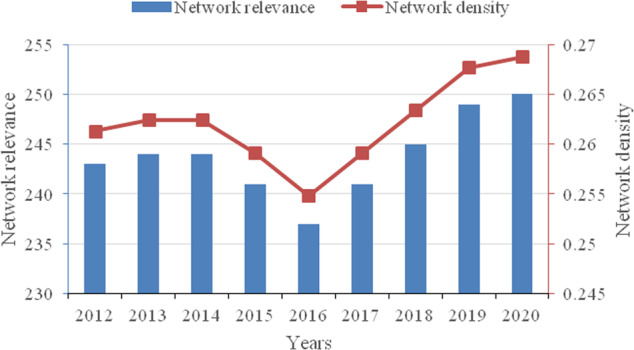
Network relevance and network density from 2012 to 2020. The number of network relationships and network density remained basically unchanged from 2012 to 2014 but declined from 2014 to 2016 and then continued to rise.

**Fig. 3:**
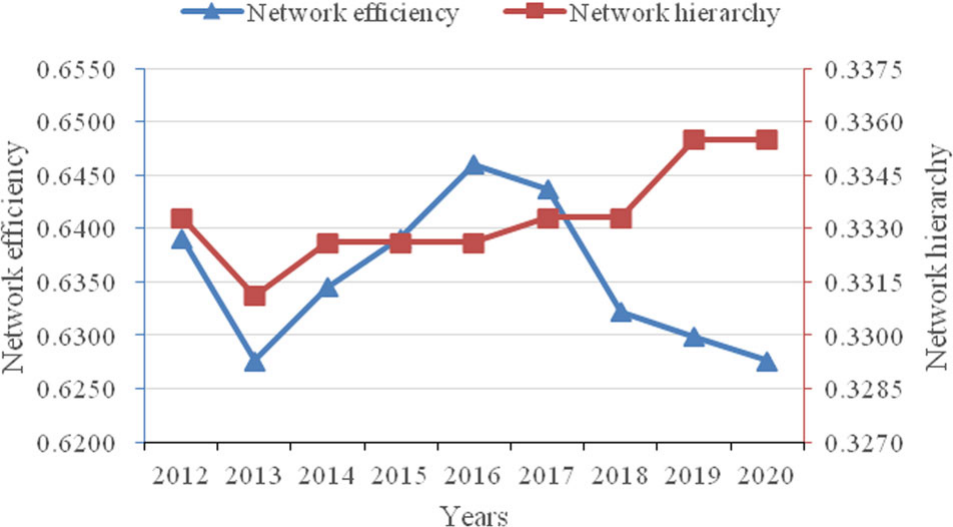
Network efficiency and network hierarchy from 2012 to 2020. The network hierarchy has shown a steady and increasing trend since 2013. Network efficiency fluctuates between 0.6276 and 0.6460, indicating that the correlations between economic resilience have multiple overlaps.

The overall correlation characteristics of the network are described in terms of network relevance, network density, network efficiency and network hierarchy. Figure 2 depicts the network relevance and network density from 2012 to 2020. Numerically, the maximum number of possible relationships in the network is 930, and the actual maximum total number of relationships is 250, with a relatively strong degree of spatial linkage. The number of network relationships and network density remained basically unchanged from 2012 to 2014 but declined from 2014 to 2016 and then continued to rise. Figure 3 depicts the network efficiency and network hierarchy. The network hierarchy has shown a steady and increasing trend since 2013. Network efficiency fluctuates between 0.6276 and 0.6460, indicating that approximately 37% of the connections in the network are redundant, i.e., the correlations between economic resilience have multiple overlaps. A possible reason for these results is that China has been increasingly active in reorienting its industrial policy to promote economic transformation and upgrading during the 12th Five-Year Plan, with 2016 having been the opening year of China’s 13th Five-Year Plan, and the country is vigorously implementing supply-side reforms to improve the adaptability and flexibility of the supply structure to changes in demand. The initial implementation of the policy will inevitably cause a considerable impact on the economic structure, which reduces the robustness of the network and makes the interprovincial linkages less tight, as shown by the low network relevance and density in 2016. After 2016, network relevance and density continued to rise, and network efficiency continued to decrease, which means that network stability increased year by year, and the Chinese economy gradually became more resilient. The network efficiency continues to decline while the network hierarchy rises, indicating that while the degree of access in the network is gradually declining, the cascading influence between nodes is increasing, forming a tight and orderly hierarchy of economic resilience.

#### Centrality analysis

This section analyzes the individual centrality in the network in 2020 by measuring degree centrality, betweenness centrality and closeness centrality indicators to examine the role of each province in the network. The measurement results are shown in Table 3.

**Table 3:** Centrality of the network in 2020.

Province	In-degree	Out-degree	Degree	Betweenness	Closeness
Beijing	15	6	53.333	3.000	66.667
Tianjin	5	3	16.667	0.046	46.154
Hebei	14	6	50.000	2.440	65.217
Shanxi	9	7	33.333	0.049	58.824
Inner Mongoria	4	8	33.333	0.594	58.824
Liaoning	2	8	30.000	1.232	56.604
Jilin	2	6	20.000	0.000	53.571
Heilongjiang	1	8	26.667	0.402	55.556
Shanghai	9	3	30.000	0.919	58.824
Jiangsu	27	4	90.000	20.441	90.909
Zhejiang	13	4	43.333	2.158	63.830
Anhui	11	6	36.667	0.787	61.224
Fujian	7	8	33.333	1.534	58.824
Jiangxi	4	8	26.667	0.033	56.604
Shandong	19	8	66.667	6.445	73.171
Henan	14	9	56.667	2.319	68.182
Hubei	16	10	66.667	4.924	75.000
Hunan	9	8	43.333	1.476	62.500
Guangdong	15	11	63.333	7.650	71.429
Guangxi	4	9	30.000	1.190	57.692
Hainan	2	3	10.000	0.000	43.478
Chongqing	7	7	33.333	0.433	55.556
Sichuan	11	11	53.333	2.460	68.182
Guizhou	4	8	26.667	0.088	56.604
Yunnan	5	8	30.000	0.205	57.692
Tibet	0	13	43.333	0.945	63.830
Shaanxi	10	12	56.667	2.539	69.767
Gansu	6	12	50.000	1.450	66.667
Qinghai	2	12	43.333	0.817	63.830
Ningxia	1	10	33.333	0.151	58.824
Xinjiang	2	14	50.000	1.319	66.667

**Fig. 4:**
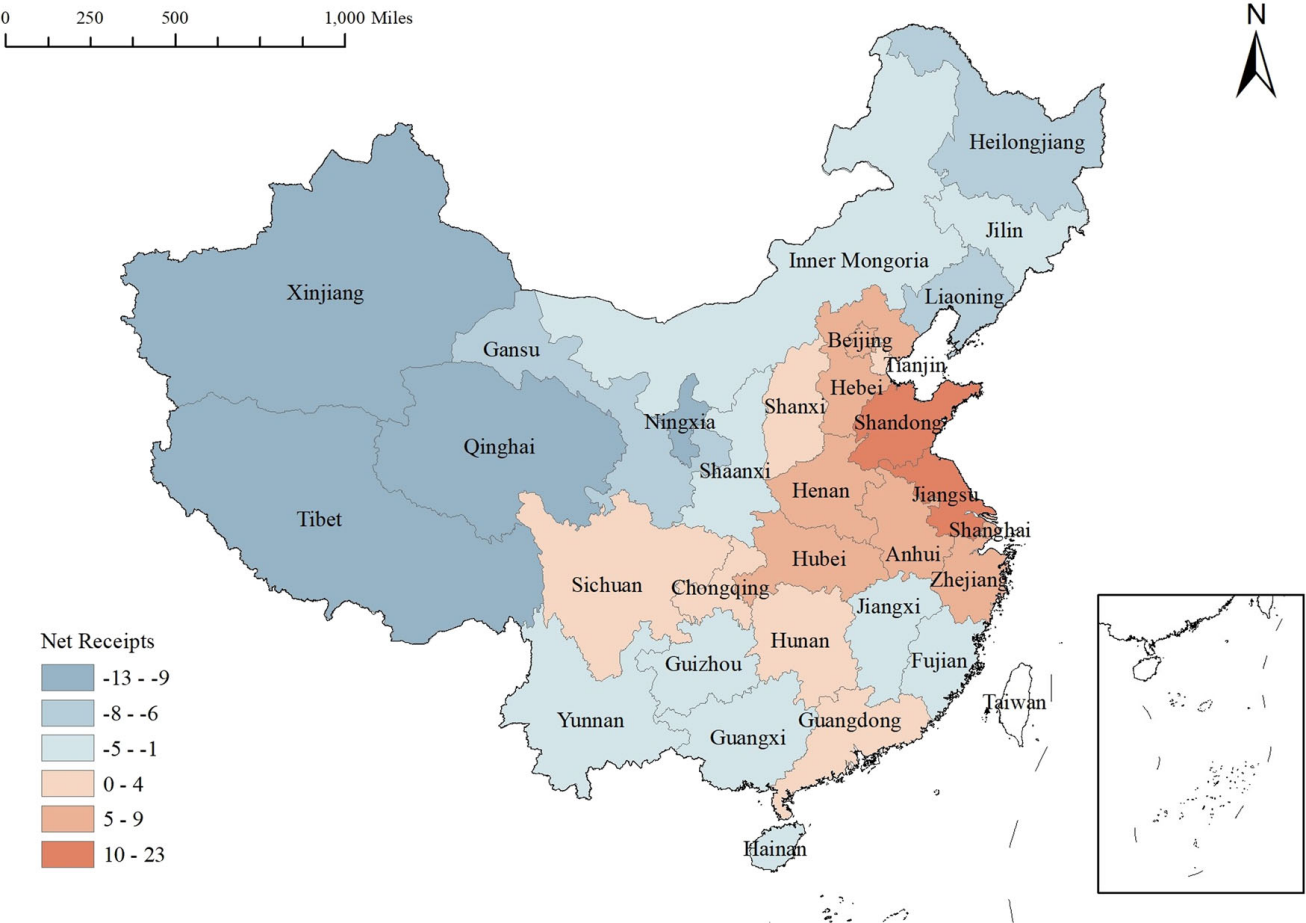
Net receipts of association relations by province in the network in 2020. The net reception number is equal to the difference between the in-degree and the out-degree.

As shown in Table 3, the mean value of the degree centrality of 31 provinces in 2020 is 41.29. Fifteen provinces have degree centrality higher than this mean value, and the top five provinces are Jiangsu, Shandong, Hubei, Guangdong, Henan and Shaanxi, which have the highest number of relationships with other provinces and have a dominant position in the network. According to the measurement results of point-in and point-out degrees in Table 3, the point-in degrees are higher than the mean value of 8.06 for 11 provinces, including Jiangsu, and the top five are Jiangsu, Shandong, Hubei, Guangdong and Beijing. The point-in degree is higher than the average value in terms of 14 provinces, including Xinjiang, and the top five are Xinjiang, Tibet, Qinghai, Gansu and Shaanxi. The higher the point-in degree of a province means that the more “resources” the province can absorb to resist external economic shocks, the more talent, capital and other factors can be gathered to recover from economic shocks as soon as possible, and the provinces that are experiencing such shocks are the “recipients” of spillover from the economic resilience of other provinces. Hubei is second only to Jiangsu and Shandong in terms of point entry. Since COVID-19 in Hubei was the most serious in early 2020, it received strong support from other provinces as well as the state in terms of policies and funds, which made Hubei passively receive more correlations within the network. In contrast, most of the provinces with higher point-out degrees have poorer economic levels and a single industrial structure, and they are “contributors” to the economic resilience of other provinces. The net receipts of each province in the network are calculated by counting the number of correlations received by the province (point-in degree) as positive and the number of correlations spilled out (point-out degree) as negative, as presented in Fig. 4. The spatial spillover direction of China’s interprovincial economic resilience is generally characterized by “west to east” and “north to south”. During the study period, this spatial spillover trend gradually increases over time, and the polarization effect is greater than the trickle-down effect in the east-central coastal region of the overall network. In addition, only Fujian has a greater point-out than point-in in the eastern and southern coastal regions, mainly due to the lack of data for Taiwan, whose economic resilience relationship with the mainland via Fujian is not reflected in the network.

The average value of betweenness centrality of 31 provinces in 2020 is 2.20. Nine provinces have betweenness centrality higher than this average, namely, Jiangsu, Guangdong, Shandong, Hubei, Beijing, Shaanxi, Sichuan, Hebei and Henan, which dominate the circulation channels of production factors and control the “exchange” between other provinces. The sum of betweenness centrality of the nine provinces above the average is 52.22, accounting for approximately 76.7%. Clearly, the betweenness centrality of each province shows obvious unbalanced characteristics, and the 9 provinces control the overall spatial association to a much greater extent than the 22 others. In contrast, Jilin and Hainan are more remote, with relatively backward economic development and a weak industrial base, and their betweenness centrality has been almost zero during the examination period, indicating their marginal position in the network and less contribution to the connectivity. It should be noted that Tibet, Gansu, Qinghai and Xinjiang have higher than average degree centrality but lower betweenness centrality, indicating that the connected routes tend to bypass the “hub” routes in the network. In contrast, Guangdong has a lower point centrality than Jiangsu, Shandong, and Hubei, but its betweenness centrality is the second highest after Jiangsu, indicating that the relatively few linkages in Guangdong are critical to the transmission of the network.

The average closeness centrality of the 31 provinces in 2020 is 62.28. Fifteen provinces have closeness centrality higher than this average, and the top five provinces are Jiangsu, Shandong, Hubei, Guangdong and Shaanxi, which are connected to other nodes through shorter paths and can be more accessible to other provinces, playing the role of central actors. Jiangsu ranks first in all three centrality degrees, indicating that Jiangsu is an irreplaceable central node in the network. Jiangsu is located in the Yangtze River Delta economic circle on the southeast coast of China, with a privileged geographical location, abundant natural resources, strong overall financial strength, and a complete industrial system with a continuously optimized industrial structure. For example, in the first half of 2021, the economy and fiscal revenue of Jiangsu recovered relatively well under the epidemic, reflecting strong economic development resilience.

**Fig. 5:**
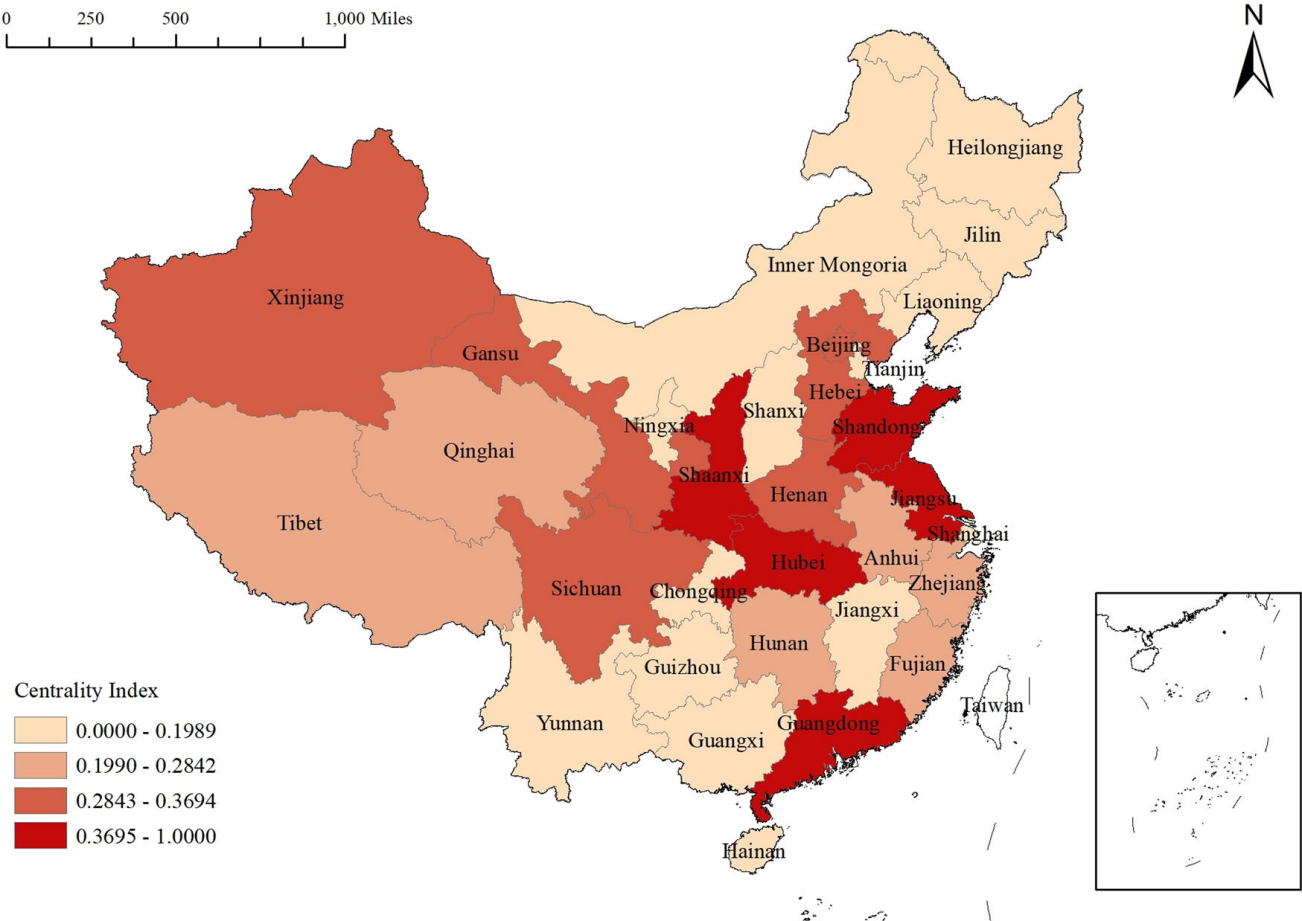
Centrality index of the network in 2020. The index is calculated from degree centrality, betweenness centrality and closeness centrality using the entropy method, and its value reflects the importance of each province for the circulation and transmission of the directed relationships in the network.

The centrality index is obtained by aggregating the degree centrality, betweenness centrality and closeness centrality through the entropy-TOPSIS method. This index does not reflect the strength of the attribute of economic resilience but rather the importance of each province for the circulation and transmission of directional relationships in the network. This paper uses 70%, 100% and 130% of the average value of the centrality index of 31 provinces in China from 2012 to 2020 as the criteria for classifying provinces into four classes: marginal, average, sub-core and core provinces, as shown in Fig. 5. The centrality indexes of the northeast and southwest provinces are low, while the centrality indexes of the southeastern coastal and central provinces are generally high. The five provinces of Jiangsu, Shandong, Guangdong, Hubei and Shaanxi are at the top of the three centrality rankings, and they are the most important agglomeration and radiation centers in the regional association framework, playing an irreplaceable penetrating role in the surrounding areas in terms of the flow of capital, scientific and technological innovation and industrial structure optimization. In addition, the centrality index of Jiangxi is significantly lower than that of the six bordering provinces in the network.

#### Spatial transfer characteristics of node centrality

Using Markov chains, the spatial transfer matrix of the centrality index of economic resilience of 31 Chinese provinces from 2012 to 2020 is obtained, as shown in Table 4. The highest values of the transfer probabilities of marginal and core provinces are on the diagonal, indicating that these two types have strong stability, with the probability of marginal provinces maintaining their original types during the study period being 81.7% and 78.6% for core provinces. In contrast, general and sub-core provinces have a high probability of shifting upward or downward. For example, the probability that a sub-core province maintains its own type is only 36.1%, and its probability of shifting upward, i.e., to a core province, is 30.6%. In general, the power floating and status adjustment of individual provinces in the network is frequent, although the overall number of network affiliations does not change very much. The government should make corresponding policy adjustments for some of the second- and third-tier provinces to increase their likelihood of moving upward.

**Table 4:** Markov transfer matrix (*k* = 4).

*t*_i_/*t*_i_ + 1	*N*	Marginal province	General province	Sub-core province	Core province
Marginal province	109	0.817	0.165	0.018	0.000
General province	47	0.404	0.319	0.234	0.043
Sub-core province	36	0.028	0.306	0.361	0.306
Core province	56	0.000	0.054	0.161	0.786

Considering that the status of a province in the network is influenced by the status of neighboring provinces, this paper uses a spatial Markov chain to construct a spatial transfer matrix to explore the transfer probability of the centrality index under the influence of peripheral regions, as shown in Table 5. The highest values of transfer probability are on the diagonal when the spatial lag is 1, i.e., the peripheral region is a marginal province, indicating that being a neighbor of a marginal province will maintain the original centrality index type of the province to a higher extent. When the spatial lag is 2, i.e., the surrounding area is a general province, the diagonal values are the lowest among all four cases, indicating that being a neighbor of a general province will maintain the province’s original type to the least extent while having a high probability of upward shift in such a case. The reason for this is that the general type of provinces can be divided into two types during the study period, either provinces that are less developed in the west but have high development potential, such as Xinjiang, Qinghai, and Tibet, or provinces that are more developed in the east and center and have their own economic growth rates, such as Anhui, Zhejiang, and Hunan. If a province is adjacent to the former, it will produce a “siphon effect” so that the province obtains more resource support; if a province is adjacent to the latter, the two provinces tend to obtain the synergistic development of complementary economic advantages and positive interaction, which will eventually increase the probability of upward transfer of the province. Overall, being adjacent to marginal and core provinces will maintain the centrality index category of the province to the greatest extent, while being adjacent to a sub-core and general provinces will give the province more opportunities to shift upward but will also bear the risk of shifting downward.

**Table 5:** Spatial Markov transfer matrix (*k* = 4).

Spatial lag	*t*_i_/*t*_i_ + 1	*N*	1	2	3	4
1	1	21	0.952	0.048	0.000	0.000
	2	3	0.000	0.667	0.333	0.000
	3	5	0.000	0.200	0.600	0.200
	4	10	0.000	0.000	0.100	0.900
2	1	32	0.750	0.219	0.031	0.000
	2	13	0.308	0.231	0.385	0.077
	3	14	0.000	0.214	0.286	0.500
	4	8	0.000	0.125	0.250	0.625
3	1	32	0.781	0.188	0.031	0.000
	2	19	0.368	0.368	0.211	0.053
	3	14	0.071	0.429	0.357	0.143
	4	22	0.000	0.091	0.227	0.682
4	1	24	0.833	0.167	0.000	0.000
	2	12	0.667	0.250	0.083	0.000
	3	3	0.000	0.333	0.333	0.333
	4	16	0.000	0.000	0.063	0.938

### Subordination analysis

**Fig. 6:**
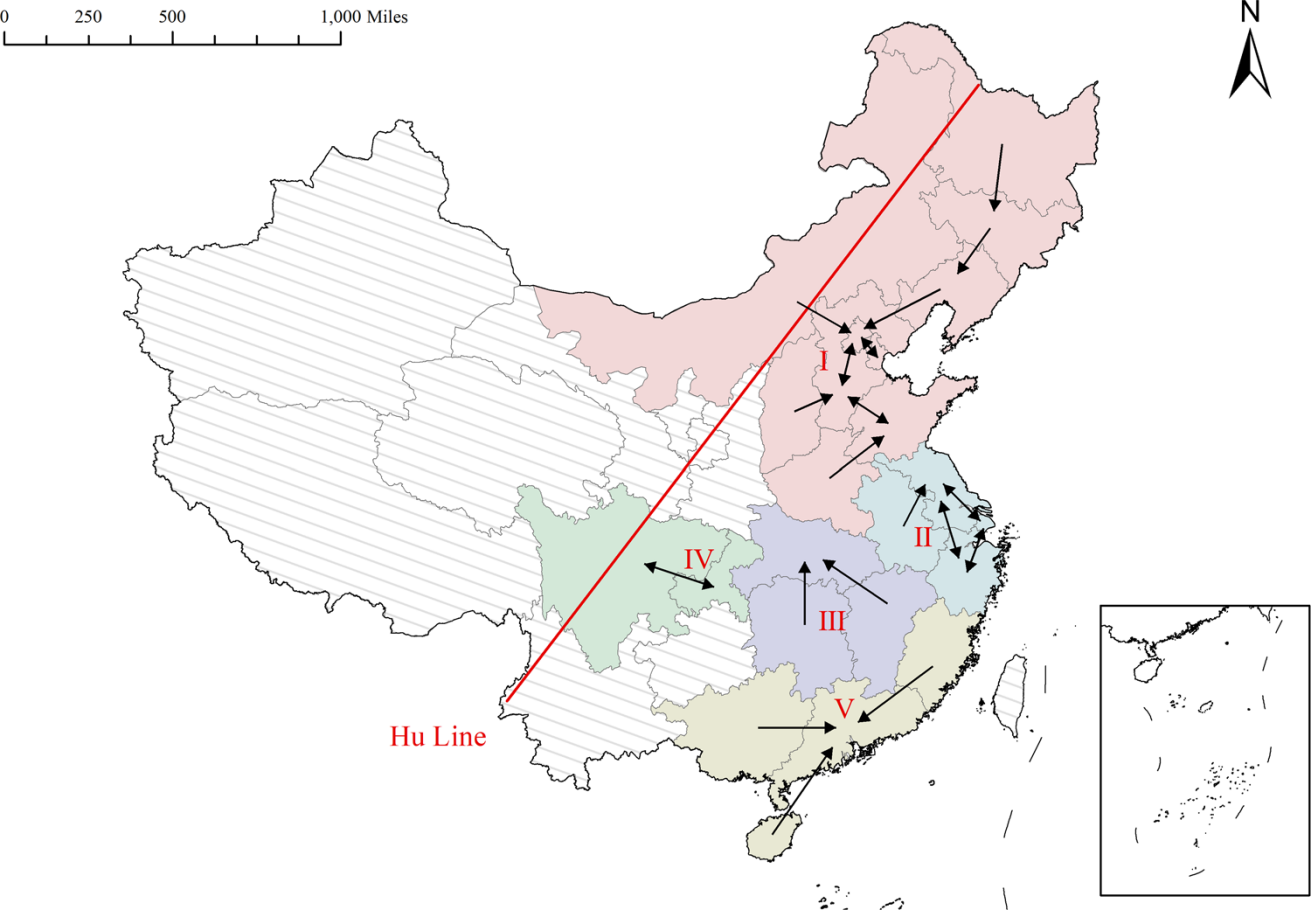
Spatial subordinated correlation network of economic resilience in 2020. The arrow indicates “subordinate”, and the node pointed by the arrow characterizes the province that receives the subordination. I–V characterize the five subgroups connected by affiliation.

This section integrates the complex correlations of the network in Fig. 1 based on the perspective of subordination, aiming to further explore the main correlation skeleton and linkage direction among individuals in the network. This paper measures the subordination of economic resilience of each province from 2012 to 2020 and visualizes the results of the subordination in 2020, as shown in Fig. 6. The Arabic numerals I–V in the figure represent the five subgroups connected by affiliation.

As shown in Fig. 6, the spatial subordinated correlation network of economic resilience has a rigid hierarchical structure, which is not characterized by a central aggregation with one or two provinces as the core but is divided into different factions in the form of subgroups with obvious geographical proximity. Eight out of 31 provinces do not form affiliation links with other provinces. In fact, the essence of the subordination linkage in the subordination association network is the aggregation of economic circles, and the higher the subordination of provinces to each other, the stronger the aggregation, and the more potential for integration.

Specifically, subgroup I, with the Bohai Sea Rim as the core and Inner Mongolia, Shanxi and Northeast provinces as the outreach, constitutes the largest group in China’s spatial subordination network. Among them, Tianjin and Beijing, Beijing and Hebei, and Hebei and Shandong are subordinate to each other, and the subordination has not been dismantled during the study period, but the extension part of this subordination association system is not stable, and the phenomenon of subordination breakage or new subordination formation will occur in certain years. This shows that the affiliation system of subgroup I is an open model of economic resilience affiliation between the core provinces and other provinces. The extensive affiliation helps spread the risk, optimize the industrial structure layout, and reduce the sensitivity of each province to the feedback of external events, i.e., improve the regional economic resilience. Subgroup II is located in the Yangtze River Delta region and consists of Jiangsu, Zhejiang, Shanghai, and Anhui, which has a stable structure of affiliation system and has not established affiliation with other provinces during the study period. Among them, Jiangsu, Zhejiang and Shanghai are mutually affiliated with each other, and their affiliation degree exceeds 0.3, which constitutes a two-way, triangular affiliation system with excellent stability. This shows that the affiliation system of subgroup II is an introverted model with strong internal affiliation and no affiliation with external provinces. A possible reason is that the Yangtze River Delta region is one of the regions with the best economic development base and the highest degree of urbanization in China. In addition, Sichuan and Chongqing, located in the upper reaches of the Yangtze River region, rely on the twin-city economic circle of the Chengdu-Chongqing region and have close internal interaction and development, forming an inseparable whole. Hunan, Hubei and Jiangxi in the middle reaches of the Yangtze River stabilized their subordination after 2016, and after the approval of the “14th Five-Year Plan” for the development of the middle reaches of the Yangtze River, these three provinces became important growth points for China’s economic resilience and important support points for the economic rise of the central region. Finally, the affiliation system of China’s economic resilience is basically distributed to the east of the Hu Line, while the provinces west of the Hu Line have vast land, sparse population and poor economic resilience, so it is difficult for them to mitigate external economic shocks or restore damaged industrial chains through the aggregation of economic circles.

### Spatial clustering analysis

Taking 2020 as an example, the block model is applied to analyze the chunking characteristics of the spatial association network of economic resilience, and the 31 provinces are divided into four plates. There are 10 provinces in plate 1: Beijing, Tianjin, Hebei, Shanxi, Inner Mongolia, Liaoning, Jilin, Heilongjiang, Shandong, and Henan; 6 provinces in plate 2: Tibet, Shaanxi, Gansu, Xinjiang, Qinghai, and Ningxia; 8 provinces in plate 3: Shanghai, Jiangsu, Zhejiang, Anhui, Fujian, Jiangxi, Hunan, and Hubei; and 7 provinces in plate 4: Guangdong, Guangxi, Hainan, Chongqing, Sichuan, Guizhou, and Yunnan.

This paper further reveals the positions of the four plates in the network through the block model, as shown in Table 6. Based on the distribution of the correlation relationships among the plates, the network density matrix of each plate is calculated, as shown in Table 7. Based on the calculations above, the network density in 2020 is 0.269, and the density matrix can be transformed into a similarity matrix by assigning the network density of the plates greater than 0.269 to 1 and the network density of the plates less than 0.269 to 0, as shown in Table 8. There is an obvious spatial spillover effect of the association relationship between the plates, but the actual proportion of the internal relationship of all four plates is higher than the expected value, indicating that the internal provinces of each subdivision are more closely connected and more related than the relationship between the plates. In the following, the network is divided into four plates: “net benefit”, “overflow”, “bilateral spillover”, and “broker”.

**Table 6:** Analysis of spillover effects.

Plate	Receive relationship		Overflow relationship		Expected internal relationship ratio	Actual internal relationship ratio
	Inside	Outside	Inside	Outside		
1	48	37	48	21	29	70
2	15	6	15	58	17	21
3	41	55	41	10	23	80
4	28	20	28	29	20	49

**Table 7:** Density matrix.

Plate	1	2	3	4
1	0.533	0.050	0.225	0.000
2	0.483	0.500	0.271	0.381
3	0.075	0.000	0.732	0.071
4	0.029	0.071	0.429	0.667

**Table 8:** Similarity matrix.

Plate	1	2	3	4
1	1	0	0	0
2	1	1	1	1
3	0	0	1	0
4	0	0	1	1

**Fig. 7:**
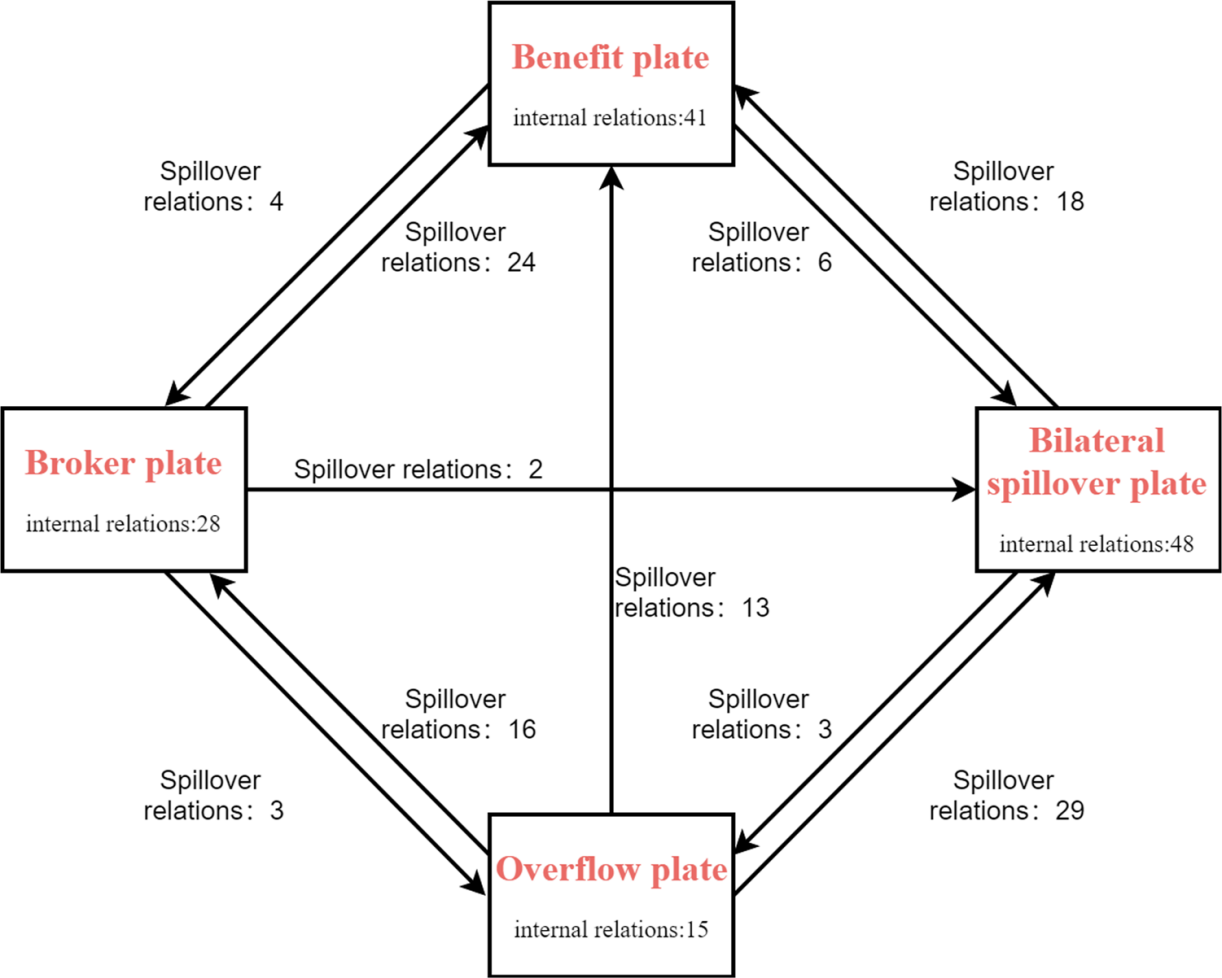
Correlation between the four blocks in 2020. The relation between provinces within a plate is called internal relation, and the relation between provinces belonging to two different plates is called spillover relation.

In the first plate, 48 relationships are inside, 37 relationships are received outside, and 21 relationships are sent outside. This plate sends out more relationships, receives more relationships and has relatively more connections between members within the plate, so the first plate is a “bilateral spillover” plate. Given the spatial subordination association network, the first plate is the same as the provinces contained in subgroup I of the subordination degree network. Within this plate, the three northeastern provinces are rich in natural resources, Hebei and Shandong have sound industrial systems, Shanxi and Inner Mongolia are rich in coal resources, and Shandong and Henan have sufficient talent reserves, while the Beijing-Tianjin-Hebei region, which is located at the core of the first plate, is the largest and most dynamic region in northern China. This indicates that the provinces in the first plate have complex internal hierarchical structures and strong economic resilience and indicates that the resilience support for other provinces outside the plate cannot be ignored, so it is a “bilateral spillover” plate. The number of relations issued outside the second plate is 58, which is much higher than the number of relations received outside the plate, and there are spillover relations to the other three plates in the similarity matrix, so the second plate is an “overflow” plate. The provinces included in the second plate have not established affiliation relationships with other provinces in the affiliation network, and they are located in the western region, which is the richest energy reserve in China. Their economic ties are more fragile and independent, and they show more resilient support to external provinces, so they are “overflow” plates. The number of relationships within the third plate is 41, and the number of relationships received outside the plate is 55, which is much higher than the number of relationships issued outside the plate, and it receives spillover relationships from the other three plates in the similarity matrix, so the third plate is a “benefit” plate. This plate contains the largest Yangtze River Delta city cluster and the middle reaches of the Yangtze River city cluster with the most growth power in China, so it is the main absorber of resources in terms of enhancing economic resilience. The fourth plate, which receives a similar total number of relationships as it sends out and is active with other plates, has an expected internal ratio of 20% and an actual internal ratio of 49%, making it a “broker” plate. Figure 7 visually depicts the correlations between the four plates.

### Factors influencing the spatial association network

*The* QAP regression analysis results are shown in Table 9. The influencing factors selected in this paper can explain 32.6% of the spatial correlation of economic resilience, and the overall fit is good. The unstandardized regression coefficients are regressed directly on the original matrix, while the standardized regression coefficients are regressed on the matrix after the standardization process. Since the standardized regression coefficients eliminate the effect of the magnitude of the observations, it is possible to compare the degree of influence of different variables on the explanatory variables based on the magnitude of the standardized regression coefficients.

**Table 9:** Results of the QAP regression analysis.

Variables	Coefficients	Sig.	Un-stdized coefficient	Stdized coefficient	Sig.
GD	0.514	0.000	0.658	0.532	0.000
OPEN	−0.136	0.007	−0.137	−0.144	0.001
DE	−0.112	0.015	−0.032	−0.035	0.202
HC	0.134	0.006	0.075	0.080	0.013
PCS	−0.135	0.006	−0.100	−0.111	0.002
TI	0.112	0.030	0.033	0.035	0.213

As shown in Table 9, the standardized regression coefficients of GD, HC, and TI are positive, indicating that these factors promote the formation of the spatial correlation of economic resilience. The regression coefficients of OPEN, DE, and PCS are negative, indicating that these factors inhibit the formation of spatial correlations of economic resilience. The analysis is as follows: first, geographical proximity passed the significance level test of 1%, probably because geographical proximity leads the cost of economic communication between provinces to be significantly lower and leads the efficiency of talent and resource flow to be significantly higher, which strengthens the correlation of economic resilience between provinces. Second, human capital passed the significance level test of 5%. Differences in economic resilience levels are usually complemented by differences in human capital, especially against the backdrop of continuously increasing urbanization disparities. The talent grabbing wars with the southeast coastal region have further led to the “plundering” of developed provinces and the “loss” of remote provinces. The southeastern coastal region is more likely to use its first-mover advantage to produce a siphon effect on population and resources, thus enhancing its own economic resilience. Third, the difference in the degree of openness passes the 1% level test. As the scale of China’s opening to the outside world continues to expand, the provinces that receive the spatial spillover relationship of economic resilience tend to be those with better economic development, while the uncertainty of import and export volume changing with tariffs, foreign exchange rates, and political protectionism has a weakening effect on economic resilience. Thus, the larger the gap between provinces in terms of import and export volumes as a share of GDP, the higher the uncertainty risk from the outside and the more impeded the transmission of resilience between provinces. Fourth, physical capital accumulation passes the 1% level test. Regions no longer single-handedly pursue rapid accumulation of physical capital in the pursuit of economic development, but they pursue more sustainable development with low capital investment, efficient capital allocation, and coordinated industrial structure. For regions that are relatively backward in development and relatively less resilient, the path dependence in the transformation process is relatively weaker and therefore more conducive to narrowing the economic resilience gap. Regarding such regions, external resources should be attracted to their own economic transformation and characteristic advantages to promote the development of economic resilience. Fifth, the variability of the digital economy and technological innovation capacity is not sufficient to reflect and explain the correlation characteristics of economic resilience, and it fails the significance test.

## Conclusions and recommendations

### Conclusions

This paper constructs a comprehensive index evaluation system for economic resilience, measures the economic resilience of 31 provinces in mainland China using the entropy method, and explores the spatial correlation characteristics of interprovincial economic resilience in China based on the spatial Markov chain, gravity model, and SNA. The main findings are as follows.

First, a tightly organized hierarchy of economic resilience emerged across China’s provinces after 2016. With the Chinese government’s structural reforms, China’s economy is gradually bursting into stronger resilience, and a tightly ordered hierarchy of economic resilience is taking shape. From the centrality analysis, we find that the spatial spillover direction of economic resilience is generally characterized by “west to east” and “north to south”. Jiangsu, Shandong, Guangdong, Hubei and Shaanxi are the top five provinces in the three centrality rankings, and they are the most important concentration and radiation centers in the regional linkage framework, playing an irreplaceable role in terms of the penetration of capital, science and technology innovation and industrial structure optimization in the surrounding areas. In addition, being adjacent to marginal and core provinces will maintain the centrality index category of a province to the greatest extent, while being adjacent to sub-core and general provinces makes the province gain more opportunities for upward shift but also bears the risk of downward shift.

Second, the spatial affiliation network of economic resilience is divided into different factions in the form of subgroups, which are basically distributed east of the Hu Line. Among them, subgroup I, with the Bohai Rim region as the core, is an open model in which the core provinces establish extensive subordinate ties with other provinces. Subgroup II, located in the Yangtze River Delta region, is an introverted model with strong internal linkages and no subordinate linkages with external provinces, while subgroup II, located in the Yangtze River Delta region, is an introverted model with strong internal ties and no affiliation ties with external provinces. These two subgroups have the most affiliations and the most typical internal structure. Thus, the essence of economic resilience subordination linkage is the convergence of economic circles and urban clusters.

Third, geographical proximity and differences in human capital drive the formation of spatial correlations of economic resilience. Differences in the degree of openness and physical capital stock hinder the linkages in existing networks. The variability in the digital economy and technological innovation capacity is not sufficient to reflect and explain the correlation characteristics of economic resilience.

### Implications

First, we should narrow the economic resilience gap between provinces and improve the balance of economic resilience. While improving the economic resilience of individual provinces, we should focus on the synergistic relationship between provinces, make full use of the radiation-driven effect of core provinces, and improve the undertaking and transfer capability of marginal provinces. Based on the different positions and roles of each province in the economic resilience network, we should continue to adjust the direction of industrial policies and promote economic transformation and upgrading by exploiting the geographical advantages and resource endowments of each province. For example, we can broaden the interaction and cooperation between the western provinces and the eastern and central provinces to build project platforms for project incubation, talent training, and market expansion. By establishing this open platform docking mechanism, the transformation of the western region to an open and sustainable type of economy is realized.

Second, we should follow the policy of regional differentiation and accurately implement policies based on local conditions. Based on the characteristics of the spatial connection of the plates, we should focus on the economic resilience of the overflow plates, and continuously strengthen the two-way spillover effect among the plates. We should promote the free flow of high-quality elements such as technology, talent and capital within the sector, strengthen the cooperation between provinces within the sector and between sectors, and constantly improve the density of the economic resilience network.

Third, as the main form of urbanization in the future, economic circles have become the most dynamic spatial organization and the most promising new pattern of development at present. Based on the existing 5 major economic resilience correlation systems, the high resilience advantages of the provinces in the Yangtze River Delta should be expanded to the west, while the stability of the northern correlators should be strengthened. The economic convergence of the Chengdu-Chongqing economic circle with its surrounding provinces should be promoted to improve the unbalanced pattern in the west.

## Supplementary information

Appendix A

## Data Availability

The datasets used or analyzed during the current study are available from the corresponding author on reasonable request.
