# Supplementary material for: Spatial association network of economic resilience and its influencing factors: evidence from 31 Chinese provinces
Source: Humanit Soc Sci Commun. 2023 Jun 5;10(1):290. doi: 10.1057/s41599-023-01783-y (PMC10243094; doi:10.1057/s41599-023-01783-y)
Supplement: Supplementary file 1 — Appendix A [file 41599_2023_1783_MOESM1_ESM.docx]

**Spatial association network of economic resilience and its influencing factors: Evidence from 31 Chinese provinces**

**Huiping Wang*, Qi Ge**

Western Collaborative Innovation Research Center for Energy Economy and Regional Development, Xi’an University of Finance and Economics, Xi’an 710100, China

Email: wanghuiping@xaufe.edu.cn (Huiping Wang) 2121011014@xaufe.edu.cn (Qi Ge)

***Appendix*** *A. Entropy-TOPSIS method*

First, the positive and negative indicators are standardized using equation (7) and equation (8) to obtain the standardized indicator $X'_{aij}$; then, the weight of indicator $j$ (equation (9)), entropy value (equation (10)), coefficient of variation (equation (11)) and weight (equation (12)) are determined, and the economic resilience $ER_{ai}$ of province $i$ in year $a(a=1,2,\cdots,n)$ is then obtained (equation (13)).

$X'_{aij}=(X_{aij}-X_{j\min})/(X_{j\max}-X_{j\min})$ (7)

$X'_{aij}=(X_{j\max}-X_{aij})/(X_{j\max}-X_{j\min})$ (8)

$P_{aij}=X'_{aij}/\sum_{a}^{s} \sum_{i}^{z} X_{aij}$ (9)

$H_{j}=-\frac{1}{\ln(ns)}\sum_{a}^{s} \sum_{i=1}^{n} P_{aij}\ln P_{aij}$ (10)

$D_{j}=1-H_{j}$ (11)

$W_{j}=D_{j}/\sum_{j=1}^{z} D_{j}$ (12)

$ER_{ai}=\sum_{j=1}^{z} W_{j}X'_{aij}$ (13)

***Appendix B****. Spatial Markov chain*

A Markov chain is a kind of stochastic process with discrete time and state. In a specific analysis, continuous data are first discretized into k types, and the distribution of the corresponding types and their changes are calculated to approximate the process of evolution. The transfer between attribute types at different moments can be represented by a k*k transfer probability matrix, and the transfer probability $m_{ij}$ is the probability that a region of type $i$ at time $t$ is transferred to type $j$ at the next moment, as shown in equation (14), where $n_{ij}$ denotes the number of regions that transfer from state $i$ to state $j$ from time $t$ to the next moment in the whole study period, and $n_{i}$ denotes the number of all regions that are in state $i$ at time $t$ in the whole study period. Using the above calculation, if the Markov transfer probability is smooth in time, we have equation (15), where $M^{s}$ is the s th power of the transfer probability matrix $M$, and $F_{t}$ and $F_{t+s}$ are the probability distributions at times $t$ and $t+s$, respectively.

$m_{ij}=\frac{n_{ij}}{n_{i}}$ (14)

$F_{t+s}=M^{s}F_{t}$ (15)

Spatial Markov chains are the product of combining the traditional Markov chain with spatial autocorrelation. The core of spatial autocorrelation analysis is spatial lag. For region $i$, its neighborhood is $j$; then, the type of spatial lag of region $i$ is determined by the spatial lag operator. As shown in equation (16), Lag is the spatial lag operator, $x_{i}$ is the attribute value of region $i$, and $w_{ij}$ is the weight. The spatial lag operator is used to determine the spatial lag type of each province, and the traditional k*k Markov matrix is decomposed into k k*k conditional transfer probability matrices, which are conditional on the spatial lag type of region $i$ at the initial moment.

$Lag=\sum_{i=1}^{n} x_{i}w_{ij}$ (16)
